# Intra-individual variability in day-to-day and month-to-month measurements of physical activity and sedentary behaviour at work and in leisure-time among Danish adults

**DOI:** 10.1186/s12889-016-3890-3

**Published:** 2016-12-03

**Authors:** E. S. L. Pedersen, I. H. Danquah, C. B. Petersen, J. S. Tolstrup

**Affiliations:** National Institute of Public Health, University of Southern Denmark, Øster Farimagsgade 5A, 2. Floor, 1353 Copenhagen, Denmark

**Keywords:** Variability, Reliability, Accelerometer, Sedentary behaviour, Physical activity

## Abstract

**Background:**

Accelerometers can obtain precise measurements of movements during the day. However, the individual activity pattern varies from day-to-day and there is limited evidence on measurement days needed to obtain sufficient reliability. The aim of this study was to examine variability in accelerometer derived data on sedentary behaviour and physical activity at work and in leisure-time during week days among Danish office employees.

**Methods:**

We included control participants (*n* = 135) from the Take a Stand! Intervention; a cluster randomized controlled trial conducted in 19 offices. Sitting time and physical activity were measured using an ActiGraph GT3X+ fixed on the thigh and data were processed using Acti4 software. Variability was examined for sitting time, standing time, steps and time spent in moderate-to-vigorous physical activity (MVPA) per day by multilevel mixed linear regression modelling.

**Results:**

Results of this study showed that the number of days needed to obtain a reliability of 80% when measuring sitting time was 4.7 days for work and 5.5 days for leisure time. For physical activity at work, 4.0 days and 4.2 days were required to measure steps and MVPA, respectively. During leisure time, more monitoring time was needed to reliably estimate physical activity (6.8 days for steps and 5.8 days for MVPA).

**Conclusions:**

The number of measurement days needed to reliably estimate activity patterns was greater for leisure time than for work time. The domain specific variability is of great importance to researchers and health promotion workers planning to use objective measures of sedentary behaviour and physical activity.

**Trial registration:**

Clinical trials NCT01996176.

## Background

Sedentary behaviour and physical inactivity have been associated with risk of adverse health outcomes and higher mortality [1–4]. In modern society, many people have office-based jobs entailing many hours of sitting [5] and hours spent sitting at work seems to be increasing [6]. Thus, occupational sitting has been the subject for much research interest lately [7]. To assess risks associated with sedentary behaviour at work and in leisure time, and due to the limitations of self-reported measures, there has recently been an increase in using accelerometers to assess sitting time, even in large population based studies. The degree to which such measurements express the true mean of an individual’s activity pattern (including both sedentary behaviour and physical activity) depends on the day-to-day and over time variability in the activity pattern. The more the activity pattern varies, the more days of monitoring are needed. In order to obtain reliable results and also keep participation burden to a minimum, an important aspect of accelerometer measurements is how many days of measurement are needed to obtain reliable estimates of sedentary behaviour and physical activity.

Several studies have examined the day-to-day variability in activity patterns in adults and found that the monitoring frame needed to reliably assess daily physical activity ranged between 3 and 16 days [8–13]. However, only few of these studies have analysed reliability for physical activity and sedentary behaviour separately and have shown opposing findings [11–13]. Further, little is known of the difference in the variability of the activity pattern at work compared to leisure time. Previously, in a study using self-reported measures, it has been shown that fewer days are needed for measuring physical activity at work (14–21 days) versus in leisure time (21–28 days) [11]. However, using objective measures, so far no studies have examined the intra-individual variability during work and leisure time, separately.

In the cluster randomized controlled trial, Take a Stand!, repeated measurements of activity were obtained via accelerometry to evaluate the effect of a multicomponent intervention among office workers on sitting time [13]. The control group comprised a perfect population for studying variability in sedentary behaviour and physical activity among office employees on weekdays.

The aim of this study was to examine the day-to-day and month-to-month variability in sedentary behaviour and physical activity during weekdays at work and leisure time in office working adults and to identify number of days needed for capturing reliable estimates of habitual physical activity and sedentary behaviour.

## Methods

### Study design

This study uses data from participants who were assigned to the control group (*n* = 135) in the Take a Stand!-trial, where accelerometer-derived measures were obtained at baseline and after 1 and 3 months.

Take a Stand! was a cluster randomized controlled trial conducted in 19 offices (clusters) in four workplaces in Denmark and Greenland from November 2013 to June 2014 aiming to reduce sitting time among office workers. The methods and primary outcomes from Take a Stand! are described in detail elsewhere [14]. In short, half of the included clusters were exposed to the intervention and the other half were included as controls. Because of the cluster design of Take a Stand! participants were recruited through their workplaces. Employees were invited by e-mail to participate, were informed of the project in writing and orally and signed informed consent forms. Eligible workplaces were office-based with employees who sat most of the workday. All participants had sit-stand desks prior to inclusion. Eligible individuals were ≥18 years, understood Danish and worked more than 4 days a week (>30 h). Exclusion criteria were sickness or disabilities affecting the ability to stand or walk, and pregnancy. Mean age was 46 years and three quarters were female (74%), which was representative of the population at the participating workplaces. Information on work environment, socio-demographic factors, health status, and health behaviour was collected by a web-based questionnaire and physical activity and sedentary behaviour were measured objectively by an accelerometer at baseline and repeated at 1^st^ follow-up after one month and at 2^nd^ follow-up after three months. The descriptive characteristics of the study sample are shown in Table 1. The trial was approved by the local Ethics Committee in Denmark (H-6-2013-005) and in Greenland (project 20914–3, id: 2014–095402) and was prospectively registered at Clinicaltrials.gov (NCT01996176). Procedures were designed in accordance with the Helsinki Declaration.

**Table 1:** Population characteristics (*n* = 153)

	Number	Percent
Demographic factors		
Age (years, mean [SD])	46	(11)
Females	100	(74)
Married/living together	111	(82)
Tertiary education	80	(59)
Health and health behaviour		
BMI (mean [SD])	26	(5)
Overweight (BMI > 25)	96	(58)
Daily smoking	23	(17)
Self-rated health		
Excellent/very good/Good	126	(93)
Self-rated fitness		
Very good/good	49	(37)

### Measurement of physical activity and sedentary behaviour

Variability in physical activity and sedentary behaviour was examined through daily means of the following variables: mean sitting time per day, mean standing time per day, mean steps per day and mean time spent in MVPA (total of walking fast, climbing stairs, running, cycling and rowing).

Participants wore an ActiGraph GT3X+ accelerometer, which records tri-axial accelerations and was set to record with 30 Hz [15]. The device is waterproof and was fixed with tape on the front of the thigh midway between the hip and knee joint. Participants wore the device 24 h a day and it was attached to each participant during working hours on Monday and removed after work on Friday. The accelerometer was removed only in case of prolonged water activities (>30 min), contact sport or skin irritation. Participants kept a log during the accelerometer period, where they recorded sleep, working hours and any irregularities or problems with the accelerometer. Data were only collected on weekdays because Take a Stand! was designed to evaluate the effect of the intervention during working hours.

Accelerometer data were processed using Acti4 software, specifically developed for thigh placement of the Actigraph [16]. Acti4 data processing has been validated in different settings, e.g. for sitting time during free living; Skotte et al. [16] found sensitivity to be 98.2% and specificity 93.3%, similar results have been found elsewhere [16–18]. Acti4 uses information on accelerometry and inclinometry to compile total minutes spent sitting/reclining, standing, walking, climbing stairs, running, cycling and rowing and all activities are thus defined as a combination of these. Activities were analysed with a minimum bout length of 15 s. for rowing and cycling, 5 s. for stair walking, sitting and lying and 2 s. for running, walking, moving and standing. As mentioned above MVPA was the total time spent walking fast, climbing stairs, running, cycling and rowing.

Time at work, during leisure time and sleep were distinguished using the information recorded in the log. Data was summed for a 24-h period, starting with work in the morning and ending with leisure time in the morning on the following day, in this way we accounted for the missing leisure time periods due to accelerometer mounting during working hours. This meant that working hours on Friday was not included in the analyses. During this 24-h period, time spent working (at the workplace or at home) was summed, equally all leisure time was summed. To be able to compare between the different days and months, work time and leisure time were standardized to eight hours per day. Eligible days included at least 4 h of work. Non-wear time was identified in three ways and data from those times were discarded, including a buffer of 10 min before and after: 1) if reported in the log; 2) if detected manually during data processing; or 3) if detected by Acti4 (a combination of > 60 min with no movement immediately preceded by strong acceleration) [16]. Only days with complete data on work and leisure time were included. If a day was excluded, the remaining included days kept their numbering in the weekly sequence to ensure that possible variance due to reactivity of wearing the accelerometer at day 1 or day-specific work/leisure structures would not disappear by mixing up the days.

### Statistical analyses

To estimate the variability in the accelerometer-derived data, random effect models were analysed using multilevel mixed linear regression. The dependent variable was the accelerometer output of interest (e.g. sitting time). Inter-individual variance (reflecting variance between clusters and between individuals) and intra-individual (residual) variance were estimated and intra-class correlation coefficients (ICC) were calculated as between subject variance/ (between subject variance + residual variance). Generally, an ICC of 0.80 is considered acceptable reliability [19]. Relative standard deviation (RSD) was estimated for the purpose of comparing total variance of the different activity variables between work time and leisure time and it was calculated as: $RSD=\frac{\sqrt{\sigma }}{\mu }$, where σ represented total variance and μ represented the mean (the constant from the regression output). Differences in variance between days and between months were estimated using likelihood ratio test comparing the full model with a model where day or month, respectively, was taken out of the model.

In order to determine the number of days needed to estimate mean sitting, standing, steps and MVPA all measurement days were combined from the three periods. Then The Spearman-Brown prophecy formula based on ICC was used to determine the number of days needed to represent each activity during working hours and during leisure time. The formula was as follows: N = [ICCd (1-ICCd)]*[(1-ICCe)/ICCe], where N = number of days needed, ICCd = desired level of reliability (0.80, 0.85 or 0.90) and ICCe = estimated level of reliability [20, 21].

Analyses were done for work time and leisure time, separately. Analyses were conducted using STATA/IC-14.0.

## Results

Table 2 shows the number of days wearing the Actigraph and the mean wear time at work and during leisure time at each of the months of measurements. Mean number of wearing days was a little higher in the second month (3.5 wearing days) compared to the first month and the third month (3.2 and 3.1 wearing days, respectively). Time spent at work varied little between the three months with a mean ranging from 446 min/day (Standard deviation (SD) = 50) to 458 min/day (SD = 51). At every measurement day and for every measurement month, participants had slightly more leisure time than working time. Generally, for both work and leisure time, wear time was lower at day 1 (where the accelerometer was put on during working hours) compared to other days.

**Table 2:** Wear time of ActiGraph accelerometer at day 1–4 of each of the three measurement months by work and leisure time

	Month 1 (*n* = 132)	Month 2 (*n* = 119)	Month 3 (*n* = 111)
	Mean (SD)	Mean (SD)	Mean (SD)
No. of wearing days	3.2 (0.8)	3.5 (0.8)	3.1 (0.9)
Median (IQR)	3 (3;4)	4 (3;4)	3 (3;4)
Work-hour, min/day			
Day 1	381 (90)	429 (99)	414 (92)
Day 2	456 (56)	459 (68)	452 (62)
Day 3	454 (65)	458 (69)	467 (78)
Day 4	487 (85)	502 (71)	473 (91)
Mean	446 (50)	458 (51)	447 (56)
Leisure-time, min/day			
Day 1	448 (88)	486 (103)	475 (91)
Day 2	518 (81)	525 (92)	541 (88)
Day 3	527 (100)	519 (91)	518 (108)
Day 4	490 (94)	475 (89)	505 (120)
Mean	502 (66)	503 (67)	514 (78)

**Fig. 1:**
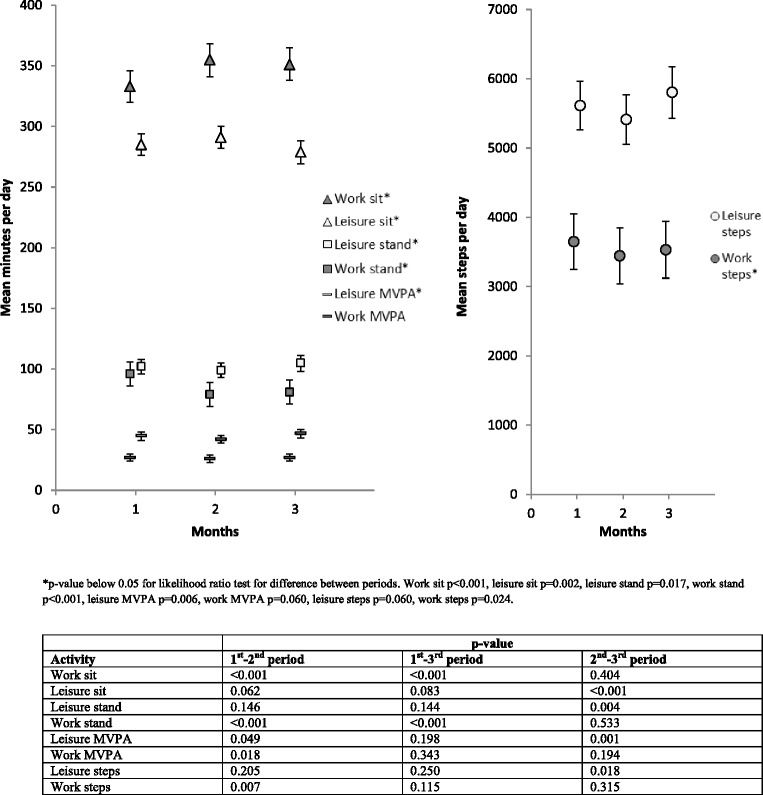
Mean minutes spent sitting, standing and in moderate-to-vigorous physical activity (MVPA) per day and mean steps at work and during leisure per day at the three measurement months

Mean time spent on sedentary behaviour and physical activity during work and leisure time by single days of measurement and by the three measurement months are shown in Table 3. Results showed no significant difference in the mean minutes/per day measured from day-to-day during work and leisure time for any of the included sedentary behaviour and physical activity variables. Also, the differences in mean minutes/day measured from month-to-month were small, although the differences were statistically different for sitting and standing during both working hours and leisure time and also for MVPA in leisure time and mean steps per day at work (Fig. 1)

**Table 3:** Minutes spent sitting, standing and in moderate-to-vigorous physical activity (MVPA) per day and steps per day during work and in leisure time at day 1–4 for month 1, 2 and 3. (*n* = number of participants with valid accelerometer information on the particular day)

	Month 1		Month 2		Month 3		*p*-value day^a^
	*n*	Mean (SD)	*n*	Mean (SD)	*n*	Mean (SD)	
Work sit							0.700
Day 1	83	326 (83)	112	359 (69)	90	356 (74)	
Day 2	127	341 (62)	116	345 (83)	107	352 (82)	
Day 3	124	345 (69)	105	351 (71)	94	356 (71)	
Day 4	93	328 (91)	87	376 (54)	52	342 (89)	
Work stand							0.656
Day 1	83	101 (72)	112	77 (60)	90	80 (61)	
Day 2	127	89 (53)	116	90 (72)	107	82 (70)	
Day 3	124	87 (58)	105	84 (61)	94	76 (59)	
Day 4	93	103 (79)	87	61 (40)	52	87 (12)	
Work steps							0.262
Day 1	83	3716 (1499)	112	3316 (1608)	90	3247 (1391)	
Day 2	127	3604 (1432)	116	3271 (1487)	107	3404 (1665)	
Day 3	124	3411 (1543)	105	3279 (1421)	94	3478 (1393)	
Day 4	93	3293 (1355)	87	3162 (1507)	52	3740 (1717)	
Work MVPA							0.314
Day 1	83	22 (11)	112	22 (12)	90	22 (11)	
Day 2	127	26 (11)	116	24 (12)	107	25 (14)	
Day 3	124	24 (11)	105	23 (11)	94	25 (11)	
Day 4	93	24 (11)	87	25 (12)	52	28 (14)	
Leisure sit							0.360
Day 1	83	282 (63)	112	291 (58)	90	284 (58)	
Day 2	127	279 (59)	116	287 (58)	107	278 (63)	
Day 3	124	285 (56)	105	293 (59)	94	276 (65)	
Day 4	93	301 (59)	87	292 (67)	52	272 (71)	
Leisure Stand							0.382
Day 1	83	100 (35)	112	99 (38)	90	103 (35)	
Day 2	127	105 (35)	116	102 (39)	107	107 (38)	
Day 3	124	103 (37)	105	99 (36)	94	106 (40)	
Day 4	93	94 (34)	87	100 (41)	52	108 (41)	
Leisure steps							0.780
Day 1	83	5900 (2986)	112	5361 (2646)	90	5555 (2623)	
Day 2	127	5770 (2730)	116	5433 (2669)	107	5542 (2533)	
Day 3	124	5669 (2634)	105	5362 (2823)	94	5932 (2919)	
Day 4	93	5065 (2230)	87	5253 (2555)	52	6407 (3637)	
Leisure MVPA							0.293
Day 1	83	45 (25)	112	43 (28)	90	44 (24)	
Day 2	127	51 (29)	116	47 (27)	107	50 (29)	
Day 3	124	48 (28)	105	44 (26)	94	52 (32)	
Day 4	93	40 (22)	87	40 (25)	52	49 (28)	

^a^Likelihood ratio test for equal means between days

Variance components (inter- and intra-individual) are shown in Table 4. Overall, the variation in sitting time did not seem to differ between work and leisure time, whereas the variation in standing time was greater at work (RSD = 0.62) compared to during leisure time (RSD = 0.38). The variation in mean steps per day and time spent on MVPA, however, was greater during leisure time compared to at work. The inter-individual variability in the activity pattern was mainly explained by variability between individuals more than between clusters. The variance explained by the difference between clusters only had an influence on physical activity at work; mean steps per day comprised 12% of the total variance and MVPA comprised 11% (data not shown). The intra-individual variability in all included activity-variables was smaller at work (50-54%) than during leisure time (54-63%).

**Table 4:** Variance component analyses of sitting time, standing time, and time spent on moderate-to-vigorous physical activity (MVPA) per day as well as for total steps per day at work, in leisure time and in total (work and leisure time combined)

Model^a^	Constant^b^	95% CI	RSD^c^	ICC^d^	Intra-individual variation^e^
work sit (min)	323	300–347	0.23	0.46	54%
work stand (min)	102	82–121	0.62	0.49	51%
work steps	3761	3211–4310	0.40	0.50	50%
work MVPA (min)	27	22–31	0.43	0.49	51%
leisure sit (min)	282	264–300	0.21	0.42	58%
leisure stand (min)	97	86–109	0.38	0.46	54%
leisure steps	5682	4919–6446	0.48	0.37	63%
leisure MVPA (min)	47	40–54	0.52	0.41	59%
total sit (min)	606	574–637	0.16	0.50	50%
total stand (min)	199	173–225	0.39	0.57	43%
total steps	9349	8357–10340	0.34	0.43	57%
total MVPA (min)	73	65–81	0.38	0.44	56%

^a^Multilevel linear regression model with the activity of interest as dependent variable adjusted for sex, age, educational level and BMI

^b^Constant representing mean value for a male participant aged 45 with tertiary education and a BMI below 25

^c^Relative standard deviation = standard deviation/mean

^d^Interclass correlation coefficient: part of total variance explained by difference between individuals

^e^Percentage of total variance explained by variation within individual

Number of measurement days needed to estimate sedentary behaviour and physical activity is shown in Table 5. The number of measurement days needed to obtain 80% reliability ranged between 4.0 for steps per day to 4.7 days for sitting time. More days were needed to reliably estimate sedentary behaviour and physical activity during leisure time, ranging from 4.7 for standing time to 6.8 days for total steps. When time was not divided into working hours and leisure time, fewer days were needed to obtain 80% reliability, ranging from 3.0 days for standing time to 5.3 days for total steps.

**Table 5:** Number of measurement days needed for 80%, 85% and 90% reliability when measuring sitting time and physical activity at work and in leisure time by using a thigh-born accelerometer (Actigraph)

	No. Days, 80% reliability	No. Days, 85% reliability	No. Days, 90% reliability
Work sit (min)	4.7	6.7	10.6
Work stand (min)	4.2	5.9	9.4
Work steps	4.0	5.7	9.0
Work MVPA (min)	4.2	5.9	9.4
Leisure sit (min)	5.5	7.8	12.4
Leisure stand (min)	4.7	6.7	10.6
Leisure steps	6.8	9.6	15.3
Leisure MVPA (min)	5.8	8.2	13.0
Total sit (min)	4.0	5.7	9.0
Total stand (min)	3.0	4.3	6.8
Total steps	5.3	7.5	11.9
Total MVPA (min)	5.1	7.2	11.5

## Discussion

In this study, we found no variation in day-to-day measurement of activity but small differences were observed for month-to-month measurements. Results suggest that during working hours, 4.7 days are needed to reliably estimate sitting time. Less monitoring time is needed for measuring physical activity at work (4.0 days for steps and 4.2 days for MVPA). During leisure time, 5.5 days are needed for estimating sitting time while more monitoring time is needed to reliably estimate physical activity (6.8 days for steps and 5.8 days for MVPA).

Although, previous findings vary somewhat between studies [8, 9, 11, 13, 21], most evidence suggest that a reliable estimate of sedentary behaviour and physical activity using an accelerometer is achieved with 3–7 days of monitoring over a single period. This is in line with the findings in the present study. Our results add to the current literature showing that the day-to-day variability of sedentary behaviour and physical activity was greater during leisure time compared to during working hours. Thus, more days are needed to obtain reliable estimates for leisure time than working hours and also more days are needed to study these two domains separately compared to total time. The difference in reliability for measurements of worktime and leisure time physical activity is important for studies focusing on health effects of domain-specific physical activity. Studies show that associations between physical activity and all-cause mortality differ between domains (work, sports, household, leisure) [22–24]. Activity was measured using questionnaires but for more valid results, physical activity should be measured using objective measures. For the purpose of designing studies like these, it is important to consider that activity patterns vary across domains and that different numbers of measurement days are needed to get reliable estimates of sedentary behaviour and physical activity, which the results of the present study show.

Activities that are less predictable on a day-to-day basis require more monitoring days to reliably predict behaviour. In the current study, we observed several important activity-specific differences. For leisure and work combined, less variability was seen for sitting and standing (3–4 days of measuring were needed for 80% reliability) compared to total steps and MVPA (requiring more than 5 days of measuring for 80% reliability). This finding is in contrast with other findings where sedentary behaviour needed more days of measuring compared to MVPA [11, 25]. In these studies, however, the populations differed in regard to age and demographics, which indicates that variability in activity depends on the characteristics of the population. Furthermore, in the present study, when separating work and leisure time, the opposite patterns of variability in activity was found for work where more days were needed to measure sitting and standing compared to steps and MVPA. This emphasizes the importance of considering type of activity as well as domain when measuring physical activity and sedentary behaviour. The contrasting domain-specific findings are in agreement with previous studies in which some have indicated that more days are needed when measuring sedentary behaviour compared to physical activity [13, 24] while others have shown the opposite [10, 12]. In line with this, it is important to be aware that variation might be different for other outcomes than the ones described in the present study (e.g. vigorous activity or sedentary behaviour defined in another way).

In the current study, we did not observe day-to-day variability, which in part could be due to the structure of the days for the participants. They all had desk-based jobs which entailed little movement during the day and job tasks did not vary across days. We observed month-to-month variability but the relative differences between the periods were small: between 5% for leisure sitting and 9% for leisure steps (data not shown). Due to the small relative difference between periods, we included days from all the three periods in the analysis of days needed to measure to obtain a certain level of reliability. The variation between the periods could be due to seasonal differences in e.g. weather and variation in work tasks over the months. Often accelerometers are only worn for a single period of time e.g. 7 days, and our results indicate that inclusion of additional measurement weeks or seasons will add variability and show that more days are needed to reliably estimate activity patterns across a period of time. Few studies have determined the reliability for several periods of measurement over the course of a year, of which all have shown considerable intra-individual variation [10, 26, 27]. However, although an increased monitoring length might improve reliability and consequently the validity of the study conclusions, the burden for study participants and study feasibility also needs to be considered.

### Strengths and limitations

Our study had strengths and limitations. One strength of the study was the use of thigh-born accelerometer that was worn for 24 h per day, which ensured no bias from difference in wear time. Secondly, by placing the accelerometer on the thigh, we were able to distinguish sitting time from standing or sleeping time. Thirdly, we included a relatively large sample of adults (*n* = 135). The study has limitations as well. Firstly, in the present study, the variability did not vary from day-to-day which has also been shown in other studies [8, 13, 25]. ICC is a relative and context-specific estimate that depends on the heterogeneity of the sample [28, 29]. The study participants were middle-aged adults with office-based work and variability in physical activity measurements may be different among other age groups or people with other types of jobs or without jobs. A second limitation was that activity measurements were only obtained on workdays; hence, we could not investigate differences in variability between work days and weekend days. In a study among 30 males it was found that habitual physical activity tended to be greater during weekdays compared to weekend days [8] but in a study by Bingham et al. [25] no difference in variance of physical activity was found between weekdays and weekend days and thus, it was not possible to conclude whether weekend days have to be included when estimating habitual physical activity. Thirdly, because no information on intensity was obtained, we could not identify vigorous physical activity from moderate-to-vigorous activity and thereby not elaborate about any possible differences in reliability between the two intensity levels.

Future studies should seek to verify the current findings of variability across populations and examine variability in sedentary behaviour and physical activity within a measurement period containing both weekdays and weekend days. Variability is a natural part of sedentary behaviour and physical activity and it should not be ignored or avoided because it has important implications for measurement and analysis. Further, it would be interesting to study variability in activity patterns, as well as mean activity across a period, in relation to physical activity and sedentary behaviour research in order to understand what role variability in activity plays in relation to health.

## Conclusions

We examined day-to-day and month-to-month variation in sedentary behaviour and physical activity in an adult population with office-based work. Overall, more measurement days were needed to reliably estimate activity patterns during leisure time compared to working hours, as leisure time activities are more variable than working activities. The more variation in activity patterns, the more days of measurements are needed to get reliable estimates. Also, the results indicate that the variability in sedentary behaviour compared to physical activity depends on the domain of interest. Findings from this study combined with previous knowledge implies that variability in activity patterns are important to regard when designing studies entailing measurements of physical activity and sedentary behaviour in order to get reliable estimates.

## Abbreviations

MVPA: Moderate-to-vigorous physical activity
N: Number of days needed
n: Number of participants
